# Estimating risk of rapid disease progression in pediatric patients with autosomal dominant polycystic kidney disease: a randomized trial of tolvaptan

**DOI:** 10.1007/s00467-023-06239-8

**Published:** 2023-12-13

**Authors:** Djalila Mekahli, Lisa M. Guay-Woodford, Melissa A. Cadnapaphornchai, Stuart L. Goldstein, Ann Dandurand, Huan Jiang, Pravin Jadhav, Laurie Debuque

**Affiliations:** 1 PKD Research Group, Laboratory of Ion Channel Research, Department of Cellular and Molecular Medicine, KU Leuven, Louvain, Belgium; 2 Department of Pediatric Nephrology, University Hospital of Leuven, Herestraat 49, B-3000 Louvain, Belgium; 3 Center for Translational Research, Children’s National Research Institute, Washington, DC USA; 4 Rocky Mountain Pediatric Kidney Center, Rocky Mountain Hospital for Children at Presbyterian/St. Luke’s Medical Center, Denver, CO USA; 5 Center for Acute Care Nephrology, Cincinnati Children’s Hospital Medical Center, University of Cincinnati School of Medicine, Cincinnati, OH USA; 6 Cerevel Therapeutics, Cambridge, MA USA; 7 Otsuka Pharmaceutical Development & Commercialization, Inc., Princeton, NJ USA; 8 MDCI Biosciences LLC, Philadelphia, PA USA

**Keywords:** Autosomal dominant polycystic kidney disease, ADPKD, Chronic kidney disease, Risk assessment, Kidney volume, Pediatric, Tolvaptan

## Abstract

**Background:**

Tolvaptan preserves kidney function in adults with autosomal dominant polycystic kidney disease (ADPKD) at elevated risk of rapid progression. A trial (NCT02964273) evaluated tolvaptan safety and pharmacodynamics in children (5–17 years). However, progression risk was not part of study eligibility criteria due to lack of validated criteria for risk assessment in children. As risk estimation is important to guide clinical management, baseline characteristics of the study participants were retrospectively evaluated to determine whether risk of rapid disease progression in pediatric ADPKD can be assessed and to identify parameters relevant for risk estimation.

**Methods:**

Four academic pediatric nephrologists reviewed baseline data and rated participant risk from 1 (lowest) to 5 (highest) based on clinical judgement and the literature. Three primary reviewers independently scored all cases, with each case reviewed by two primary reviewers. For cases with discordant ratings (≥ 2-point difference), the fourth reviewer provided a secondary rating blinded to the primary evaluations. Study participants with discordant ratings and/or for whom data were lacking were later discussed to clarify parameters relevant to risk estimation.

**Results:**

Of 90 evaluable subjects, primary reviews of 69 (77%) were concordant. The proportion considered at risk of rapid progression (final mean rating ≥ 3.5) by age group was: 15–17 years, 27/34 (79%); 12– < 15, 9/32 (28%); 4– < 12, 8/24 (33%). The panelists agreed on characteristics important for risk determination: age, kidney imaging, kidney function, blood pressure, urine protein, and genetics.

**Conclusions:**

High ratings concordance and agreement among reviewers on relevant clinical characteristics support the feasibility of pediatric risk assessment.

**Graphical abstract:**

A higher resolution version of the Graphical abstract is available as Supplementary information

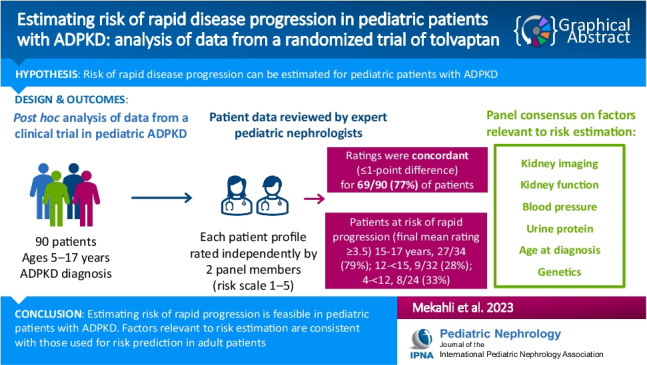

**Supplementary Information:**

The online version contains supplementary material available at 10.1007/s00467-023-06239-8.

## Introduction

Autosomal dominant polycystic kidney disease (ADPKD) has historically been considered as an adult disease [1]. Rates of cyst progression leading to chronic kidney disease are highly variable, however, with approximately 3% of children who carry ADPKD-causing mutations experiencing either early-onset or unusually progressive disease [2]. Moreover, there is an increasing awareness that, even in patients who are asymptomatic during childhood, the disease manifests in detectable ways. Cyst formation and expansion initiate early in life and affect kidney function before any decline in glomerular filtration rate (GFR) occurs [3]. Reduction in urinary concentrating capacity is one of the earliest signs of ADPKD observed in children and is present in 58% of the affected pediatric population [4–6]. Twenty percent of pediatric patients with ADPKD exhibit proteinuria, and glomerular hyperfiltration has been described in 18–21% [6, 7]. Disease progression may be reflected in extra-renal complications, with a reported hypertension prevalence of 20% in the affected pediatric population versus 2% in this age group among unaffected individuals. Although symptoms are less common in children and adolescents than adults, some pediatric patients report cyst-related pain [6]. In general, symptoms and psychological impacts of early-stage ADPKD are underestimated by clinicians [8].

Given that ADPKD is now understood as a longitudinal disease starting in childhood, disease-modifying treatment may exert the greatest benefit if initiated before most of the intact kidney parenchyma has been lost, while kidney function is maintained. Time to kidney failure would thus be delayed as compared to later intervention.

Tolvaptan is available for the treatment of ADPKD to delay decline in kidney function in adults (≥ 18 years) at risk of rapid progression [9]. A clinical trial (EudraCT number: 2016–000187-42; ClinicalTrials.gov identifier NCT02964273) evaluated the safety and pharmacodynamics of tolvaptan in children and adolescents with ADPKD [10, 11]. The study protocol and the first results of the randomized, double-blind phase have been reported [10, 11]. As validated diagnostic criteria for childhood ADPKD are lacking [1, 12], the presence of ADPKD was determined in the study subjects using criteria determined by expert consensus [10].

A possible extension of the adult indication to pediatric patients presupposes that children at risk of rapid progression can be identified. Tools exist for the identification of adult patients at elevated risk of rapid progression but cannot be applied to the pediatric population. The ADPKD risk classification algorithm developed by Mayo Clinic researchers is validated only for patients ages 15 years and over [13]. The Predicting Renal Outcomes in ADPKD (PROPKD) score cannot be calculated for patients age < 35 years in the absence of hypertension and onset of first urologic event [14]. Genetic assessment alone cannot be used to determine risk of rapid progression. Although ADPKD is known to be caused by mutations in the *PKD1* or *PKD2* genes, the genotype–phenotype relationship is complex, with a high amount of allelic heterogeneity [15]. In the absence of a validated risk stratification system, the tolvaptan pediatric study enrolled a broad population of subjects diagnosed with ADPKD, and risk of rapid progression was not part of the eligibility criteria [10]. To determine whether risk of rapid disease progression in pediatric ADPKD can be assessed and to identify parameters most relevant to risk estimation in this population, an expert panel was convened to retrospectively evaluate the baseline characteristics of patients enrolled in the tolvaptan pediatric study.

## Methods

### Design

As reported, key enrollment criteria for the tolvaptan pediatric study were ages 4 to 17 years (inclusive) and a diagnosis of ADPKD [10]. Diagnosis was based on family history and/or genetic evidence in conjunction with the confirmed presence of kidney cysts during study screening. Specifically, subjects aged 12–17 were required to have ≥ 10 kidney cysts on MRI, each cyst measuring ≥ 0.5 cm. Those aged 4–11, who did not undergo MRI, were required to have ≥ 4 kidney cysts on ultrasound, each ≥ 1 cm. Subjects also had to have eGFR ≥ 60 mL/min/1.73 m^2^ by the bedside Schwartz formula [16].

Assessments performed at study screening and/or baseline visits included vital signs, serum chemistry, urinalysis, medical history, MRI/ultrasound imaging, and if available, ADPKD genetic results. The screening and baseline visits were to be made within 31 days of each other. For the present analysis, subject data from the screening/baseline assessments were retrospectively evaluated.

The study sponsor convened a panel of four expert pediatric nephrologists (MC, SG, DM, LGW) to review the subject data and determine the risk of rapid progression for each subject. Data on each of the 91 enrolled subjects was extracted from the study database and placed into a separate document for each subject for distribution to panel members using a secure data transfer portal. Initially, a pilot review was conducted in which the patient profiles of six study subjects were distributed to three of the reviewers to test the distribution process and assess review parameters. The reviewers were asked to review each subject on a 5-point scale, with a score of 1 denoting that the subject is “least likely to be a rapid progressor” and 5 denoting that the subject is “most likely to be a rapid progressor”. Reviewers were also asked to consider the feasibility of the 5-point scale, how to describe the level of risk that should be assigned to each score (e.g., 1 – unlikely, 2 – possible, 3 – probable, etc.), and to describe the rationale for the ratings assigned to each subject. A meeting with the reviewers was subsequently held to compare the ratings assigned by each reviewer and to discuss the appropriateness of the process and parameters used. This pilot also dismissed the need to consider a 7-point scale, as all reviewers unanimously agreed to the sufficiency of the 5-point scale for pilot cases.

Following feasibility as determined in the pilot review, review and scoring of patient profiles for all enrolled subjects was conducted. There were three primary reviewers, and the fourth reviewer provided additional review for discordant cases. Each of three primary reviewers received a portion of the 91 profiles (60–61 profiles for each reviewer), so that each profile would be independently reviewed and scored by two primary reviewers in blinded fashion. The ratings assigned by each of the two primary reviewers were compared by the study sponsor. For study subjects for whom the assigned ratings were discordant (defined as a difference of ≥ 2 points between reviewers), the fourth panel member would provide an additional, secondary review and rating while blinded to the primary evaluations. The results of this additional review were recorded separately. This same panel member would also provide a blinded, independent rating for cases in which one of the primary reviewers believed there would be value to having another expert review of any subject and requested such additional input.

Finally, a panel meeting of the expert reviewers and study sponsor was held to discuss the ratings and compare the scores given by different reviewers, identify the key characteristics of patients at risk for rapid progression, and determine the final individual reviewer ratings.

### Outcomes

The number and percentage of concordant ratings assigned by the blinded primary reviewers were determined and interrater agreement was statistically evaluated as described below. Another outcome of interest was the key parameters for assessing risk of rapid progression that were identified by the expert panelists during the discussion. Potential key parameters considered for estimating risk of rapid progression were based on the literature and the panelists’ clinical experience (Table 1) [6, 13, 15, 17–21].

**Table 1:** Potential key parameters for estimating risk of rapid progression in pediatric ADPKD [6, 13, 15, 17–21]

Progression factor	Strengths	Limitations
Increased kidney volume	• Predicts rapid progression in adults [13] • Mayo imaging classification for adults can be adapted to younger patients [17]	• Mayo imaging classification is not applicable to children aged 15 years or under [13] • Lack of validated kidney dimension and volume standards adjusted for age, height, weight, and sex in the pediatric population [22]
Decreased kidney function	• Kidney function decline may occur during childhood in the most rapidly progressive forms of ADPKD [23, 24]	• Not a good risk indicator in most children with ADPKD, however, as kidney function is usually preserved [23, 24] • Decline in GFR can be masked by glomerular hyperfiltration [25]
Hypertension	• Presence of hypertension correlates with more rapid progression to kidney failure in adults [26]	• Data on the relationship of hypertension to ADPKD progression are lacking in children, but a strong positive correlation with greater kidney volume has been shown [22, 27, 28]
Albuminuria/proteinuria	• Proteinuria is correlated with more rapid progression to kidney failure in adults [29–31]	• Data are lacking for children with ADPKD [22]
Genetic	• Mutations (e.g., *PKD1* vs. *PKD2, PKD1* truncating vs. nontruncating) correlate with risk of rapid progression [32, 33]	• Genotype–phenotype relationships are complex and incompletely characterized; estimation of progression risk from genetics alone is not yet feasible [15, 22]
Glomerular hyperfiltration	• Associated with more rapid kidney growth and decline in kidney function in children with ADPKD [3]	• No unified definition of hyperfiltration [22]
Symptoms	• Symptoms are present in substantial percentages of patients with pediatric ADPKD, including macroscopic hematuria, pain, urinary concentrating deficits, and urinary tract infection [6]	• Few data on the long-term prognostic value of these symptoms in children with ADPKD [22]
Overweight/obesity	• Correlation with ADPKD progression shown for adults [18]	• Data are lacking for children with ADPKD [22]
Biomarkers^a^	• Several biomarkers correlate with ADPKD progression in adults (e.g., plasma copeptin, urinary MCP-1) [19, 20], or with CKD progression in children (urinary EGF) [21]	• Data are lacking for children with ADPKD
Age	• Earlier onset of other risk factors suggests more rapidly progressive disease	

^a^Biomarkers were not available for evaluation of progression risk in this study but are listed here as a potential progression factor for completeness

*ADPKD* autosomal dominant polycystic kidney disease, *CKD* chronic kidney disease, *EGF* epidermal growth factor, *GFR* glomerular filtration rate, *MCP-1* monocyte chemoattractant protein-1

For subjects who were assigned discordant ratings and/or had their ratings adjusted during the group discussion due to inadequate or missing data, the participants explored strategies for risk assessment when only limited information is available. Examples are presented to illustrate the factors considered by the panelists to be most important in assessing such cases.

### Statistical analyses

Interrater agreement among the primary reviewers was assessed using the Bland–Altman method [34]. Final overall ratings were determined by weighting the rating of each primary rater equally, and in cases where an additional, secondary review was provided by the fourth panel member, the secondary rating was weighted at 1.5 to provide a clear tie-breaker: [(1x + 1x)/2 OR (1x + 1x + 1.5x)/3.5)].

## Results

### Analysis population

As reported, 91 subjects with ADPKD aged 5–17 years were enrolled. In the overall study population and within age subgroups (12–17 years, 4–11 years), study subjects exhibited wide ranges for height-adjusted total kidney volume and estimated glomerular filtration rate, suggesting a broad spectrum of ADPKD severity among the enrolled subjects (Table 2). Diagnostic evaluation included genetic testing in 27/91 (30%) of subjects.

**Table 2:** Baseline demographic and clinical characteristics of the study population, overall and by age group

	Overall (*N* = 91)	Aged 12–17 Years (*n* = 66)	Aged 5–11 Years (*n* = 25)
Age (years)			
Mean (SD)	12.9 (3.0)	14.4 (1.6)	9.0 (1.9)
Female, *n* (%)	44 (48)	34 (52)	10 (40)
Race, *n* (%)			
White	88 (97)	65 (98)	23 (92)
Black	1 (1)	1 (2)	0
Asian	2 (2)	0	2 (8)
Ethnicity, *n* (%)			
Hispanic or Latino	2 (2)	2 (3)	0
Not Hispanic or Latino	89 (98)	64 (97)	25 (100)
Weight (kg), mean (SD)	52.6 (16.8)	59.0 (13.3)	35.6 (12.7)
Range	20.7, 108.2	30.4, 108.2	20.7, 74.0
Height (cm), mean (SD)	159.9 (17.3)	167.8 (10.6)	138.9 (13.7)
Range	113.0, 193.0	141.0, 193.0	113.0, 166.0
Body mass index (kg/m^2^), mean (SD)	20.0 (3.9)	20.8 (3.7)	17.9 (3.5)
Range	14.2, 34.9	14.2, 34.9	14.4, 26.9
Diagnosis age (years), mean (SD)	6.4 (5.4)	7.7 (5.3)	2.9 (3.9)
Range	0, 17	0, 17	0, 11
Genetic testing performed, *n* (%)	27 (30)	20 (30)	7 (28)
Other blood-related family with ADPKD, *n* (%)			
Yes	82 (90)	57 (86)	25 (100)
No	8 (9)	8 (12)	0
Unknown	1 (1)	1 (2)	0
Aware of family history before diagnosis, *n* (%)	82 (90)	58 (88)	24 (96)
Reason for diagnosis, *n* (%)			
Consequence of ADPKD signs or symptoms	23 (25)	17 (26)	6 (24)
Incidental (due to tests unrelated to ADPKD or its symptoms)	15 (17)	12 (18)	3 (12)
Asymptomatic screening (no prior ADPKD symptoms)	52 (57)	36 (55)	16 (64)
Height-adjusted total kidney volume (mL/cm),^a^ *n*		57	15
Mean (SD)		3.1 (3.2)	2.2 (1.3)
Range		1.5, 25.4	1.0, 5.9
eGFR by Schwartz formula, mL/min/1.73 m^2^, mean (SD)	99 (17.4)	99 (15.2)	100.3 (22.5)
Range	59.3, 159.9	69.1, 136.3	59.3, 159.9
ADPKD medical history, *n* (%)^b^			
Hepatic cysts	6 (7)	6 (9)	0
Non-hepato-renal cysts	1 (1)	1 (2)	0
Gross hematuria	3 (3)	3 (5)	0
Upper urinary tract infection	4 (4)	4 (6)	0
Proteinuria	14 (15)	12 (18)	2 (8)
Hypertension	21 (23)	16 (24)	5 (20)
Kidney pain	11 (12)	9 (14)	2 (8)

^a^Height-adjusted total kidney volume as measured using magnetic resonance imaging in the 12–17-year age group and ultrasound in the 5–11-year age group

^b^There were no subjects with a medical history of nephrolithiasis or vascular/cardiac abnormalities

*ADPKD* autosomal dominant polycystic kidney disease, *eGFR* estimated glomerular filtration rate

### Pilot evaluation

The process and parameters used were confirmed to be appropriate, and the team agreed to proceed with a full patient profile review of all 91 subjects and use the 5-point scale.

### Full study population review

**Fig. 1:**
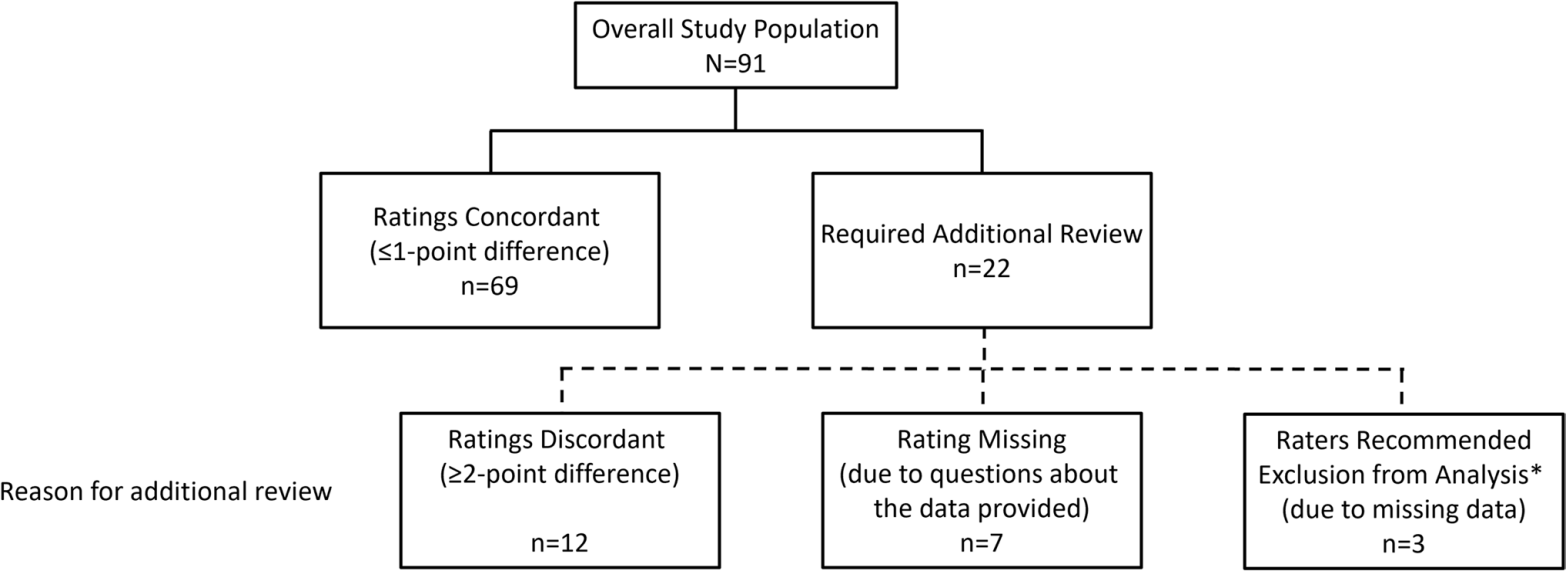
Results of ratings by the 2 initial reviewers. *These subjects were later, during the panel discussion, deemed evaluable for progression risk based on additional kidney volume data provided at the meeting and were assigned ratings for progression risk at that time. Because reviewers initially recommended exclusion of these 3 subjects from risk assessment, they are excluded from the analysis of interrater agreement

From a total of 91 subjects with ADPKD, 22 required an additional review by an independent panelist due to discordant primary ratings (≥ 2-point difference; *n* = 12), one of the primary raters not providing an initial rating due to questions about the data provided (*n* = 7), or the reviewers recommending subjects for exclusion from review (*n* = 3) because of missing kidney volume data at screening and baseline (Fig. 1). During the panel discussion, postbaseline kidney volume data were provided for the 3 subjects recommended for exclusion due to missing screening and baseline kidney volumes. The expert reviewers agreed that the 3 subjects could be assigned ratings for risk of progression based on the additional data, and all raters considered these 3 subjects to have large kidney volumes and to be at high risk of rapid progression. All panelists agreed that another of the 22 subjects requiring additional review should be excluded from risk assessment due to a history of suspected ureteropelvic junction obstruction, yielding a consensus that 90 of the 91 subjects were evaluable for progression risk.

Of the 90 subjects deemed evaluable, panelist reviews of 69 cases (77%) were concordant (≤ 1-point difference) in the first round and did not need an additional independent and blinded review. Individual reviewer ratings were adjusted for 11 subjects during the discussion to assign final ratings, including subjects for whom the primary ratings were and were not concordant. Reasons for the rating changes were based on missing or misunderstood patient data or data entry issues in assigning the ratings, including transcription errors by the reviewer, listing more than one rating score by reviewer (e.g., “a 4 or a 5”), and not providing a rating due to questions about the data (these were resolved by answering the questions and the reviewer providing a rating).

**Fig. 2:**
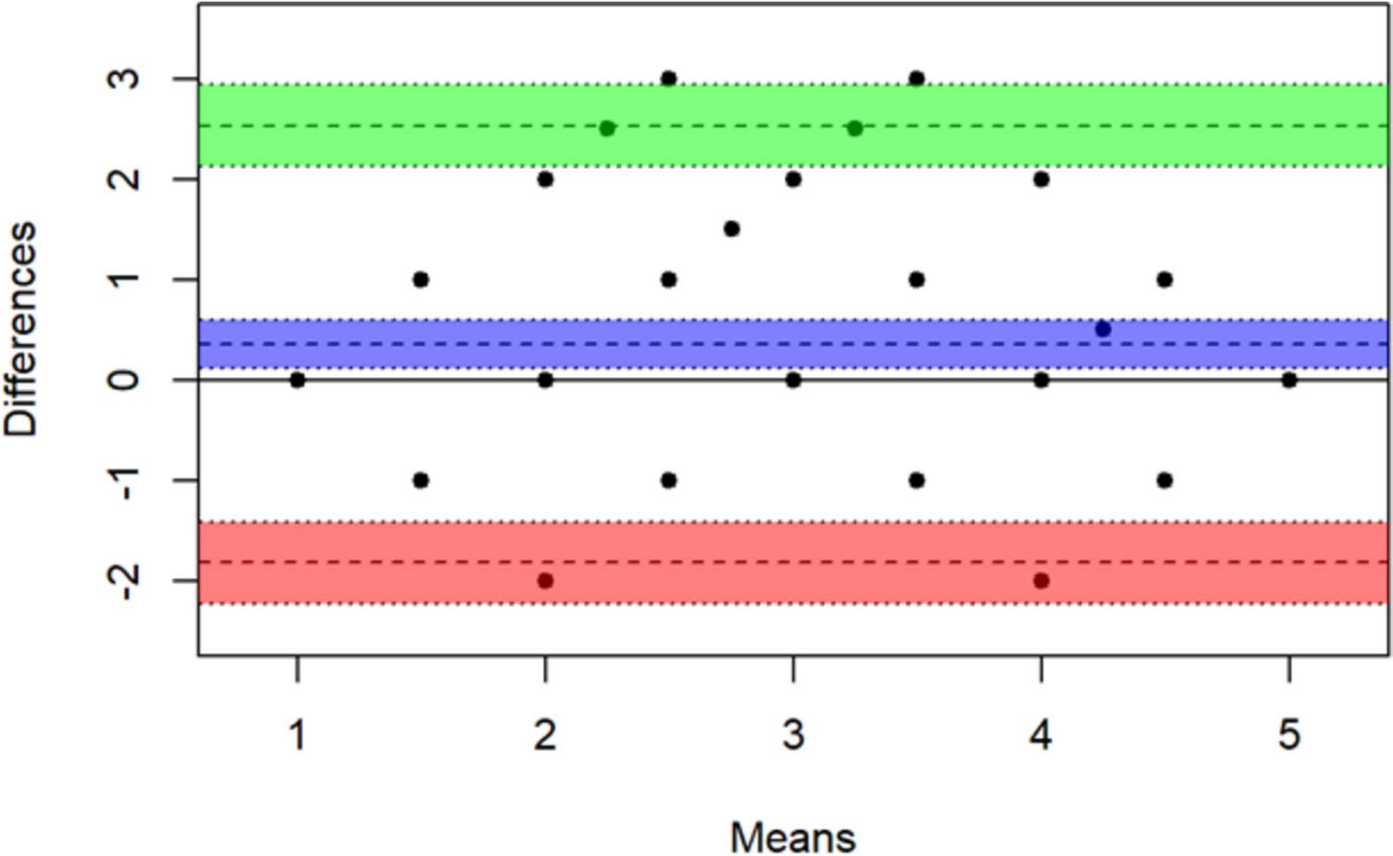
Bland–Altman plot of interrater agreement among the 2 initial reviewers

Results of the Bland–Altman analysis to assess interrater agreement among the blinded primary reviewers for the 87 subjects who were included in the primary review are shown in Fig. 2. This group consisted of the 91 total subjects less the 3 initially recommended for exclusion from the analysis due to missing data and the 1 deemed not evaluable by the panel because of suspected ureteropelvic obstruction. The mean of the two ratings for a subject is plotted on the x-axis and the difference between the two ratings is plotted on the y-axis (the plots do not distinguish identical scores). The middle line is the mean difference between ratings for all subjects (0.356 points) with a 95% confidence interval (CI). Using standard limits of agreement (i.e., ± 1.96 × standard deviation of observed differences) [34], indicated by the upper and lower lines (with 95% CI), only 2 points exceeded the 95% CI of the upper limit, and the other points were all within the limits of agreement. Thus, there was minimal evidence that scores of the primary raters differed significantly.

**Table:** 

	Estimate	Lower 95% CI	Upper 95% CI
Mean bias	0.356	0.120	0.593
Lower limit of agreement	–1.819	–2.225	–1.413
Upper limit of agreement	2.532	2.126	2.938

*CI* confidence interval

The number and percentage of subjects considered at risk of rapid progression (final weighted mean score of at least 3.5) by age group was: 15 to 17 years, 27/34 (79%); 12 to < 15 years, 9/32 (28%); 4 to < 12 years, 8/24 (33%). Twenty-five of 34 (74%) subjects ≥ 15 to 17 years of age were classified as Class 1C–1E using adult ADPKD risk classification (i.e., Mayo classification), which can be applied in that age group [13]. In that age group, an additional 2 subjects were judged to be at risk of rapid progression in the present study due to eGFR status despite being assigned to Class 1A and 1B.

In addition to genetic data, the panelists reached agreement on key phenotypic characteristics relevant to risk determination (in no particular order of importance): kidney imaging including volume, kidney function, blood pressure, and urine protein, with earlier age at onset and/or worsening of each factor indicating greater ADPKD progression risk. Several cases in which data were lacking or ambiguous and required panelist discussion about how to best assess progression risk are provided in Online Resource 1 to illustrate how the panelists resolved cases for whom incomplete information was available.

## Discussion

In this retrospective assessment for risk of rapid progression in pediatric patients with ADPKD, an expert review panel produced concordant initial risk ratings for 77% of the study participants, with a strong level of interrater agreement. This result supports that a subpopulation of children with ADPKD who are at high risk of rapid progression can be identified based on previously reported adult and/or pediatric risk factors and panel expertise. Even for cases in which all the desired information for risk assessment was not available, the reviewers were able to reach agreement on the risk level and which variables were most important. For both adults and children with ADPKD, it is common to encounter situations in the real-world clinical setting in which prognostic data are lacking [35].

It is reassuring that for the 15–17 age group, in this exercise 27/34 (79%) subjects were considered at risk of rapid progression, which is similar to the 25/34 (74%) that would be classified as high risk based on total kidney volume risk classification for patients with ADPKD aged 15 years and older [13]. The smaller proportions of participants in the younger age groups who were categorized as high risk (28% for ages 12 to < 15 years and 33% for ages 4 to < 12 years) may not reflect the true percentages of high-risk participants in those age strata; risk of progression may be more difficult to determine at younger ages with fewer or less advanced clinical disease manifestations. Even with this caveat, a notable finding was that approximately one-third of subjects aged 4 to < 15 years were evaluated as at high risk of rapid progression. In the youngest cohorts of individuals with ADPKD, a substantial proportion already has evidence of aggressive disease and will require more intensive clinical management in the opinion of reviewers and based on the data provided. Accordingly, in pediatric nephrology practice, individual disease phenotype should be considered more important than patient age.

The choice of age groups for categorization in this analysis (4 to < 12 years, 12 to < 15 years, and 15 to 17 years) was based on the age-appropriate imaging methodology for assessing kidney volume in the trial (i.e., MRI in subjects aged 12 to 17 years and ultrasound in those aged 4 to < 12 years) as well as the 3 age categories used in the evaluation of tolvaptan for pediatric use by regulatory authorities.

The panelists agreed that key characteristics relevant to risk determination were: kidney imaging, kidney function, blood pressure, urine protein, and genetics, with earlier age of onset and/or worsening of the clinical manifestations indicating higher ADPKD progression risk. These markers are qualitatively consistent with diagnostic criteria for adults [13, 14, 36]. As treatment for pediatric ADPKD evolves, including the potential use of disease-specific therapy in this population, accepted criteria for risk assessment will likely be needed to guide insurance reimbursement decisions.

Assigning weights to the respective progression risk factors identified in this study and developing a formal risk scoring system will require large-scale, longitudinal, and comprehensive assessment of pediatric ADPKD populations. Even if evaluation for risk of progression is systematized, the endpoints chosen for outcome assessment in ADPKD have been inconsistent across studies conducted in adults as well as in children and will need to be harmonized in the future [37]. At present, kidney volumetric measurement is the most promising method for accurate risk assessment. Three-dimensional ultrasound imaging methods suitable for children and a pediatric risk classification system adapted from Mayo Imaging Classification were recently shown to be feasible, although they require validation [17]. Further, more research on normal pediatric kidney dimensions, volume, and growth is needed to understand kidney growth in pediatric ADPKD [17, 22]. The presence of hypertension and urine protein are good indicators of progression risk and should be monitored [22, 38]. Decreased kidney function is a strong indicator of rapidly progressive disease, but in most pediatric patients, function is preserved, which limits the predictive utility of this risk factor [38]. Further, the applicability of the most commonly used equation for estimating eGFR in children (CKiD) has been questioned for the ADPKD population [39]. Although *PKD1* truncating and nontruncating variants are known to be associated with greater risk of rapidly progressive ADPKD, genetic determinants are too complex to accurately predict disease course [22]. Risk prediction is likely to be enhanced by biomarkers, some of which have demonstrated predictive value in adult ADPKD (plasma copeptin, urinary monocyte chemoattractant protein-1), and which require research in children [19, 20, 36, 38]. Kidney cyst quantification or textural analysis to detect structural changes in cystic or noncystic tissue, enhanced by artificial intelligence/machine learning methods, may enhance the prognostic value of imaging [36, 38].

Limitations of this research include that the data analyzed were obtained from a study of participants under the care of academic pediatric nephrology centers and therefore likely to be more serious cases and not representative of the ADPKD population as a whole. Further, the retrospective analysis reported here was not designed to generate a formalized, validated assessment system, but to demonstrate that children with ADPKD who are at increased risk of rapid progression can be identified based on clinical judgment and to specify the most relevant parameters for determining increased risk. Given the continuing challenges to diagnosis, prognostic assessment, and treatment decision-making in ADPKD, the present research advances the understanding of this condition in the affected pediatric population despite caveats to the findings.

The results of this analysis indicate that assessment of risk for rapid progression is feasible in pediatric patients with ADPKD and that the key parameters for risk assessment identified by expert consensus are consistent with those used in adult risk assessment. As recognition of ADPKD as a disease affecting the pediatric population increases, differentiated assessment of individual phenotype, regardless of patient age, will assume greater importance in clinical management.

### Supplementary Information

Below is the link to the electronic supplementary material.

Graphical abstract (PPTX 83.1 KB)

Supplementary file2 (DOCX 33.5 KB)

## Data Availability

To submit inquiries related to Otsuka clinical research, or to request access to individual participant data (IPD) associated with any Otsuka clinical trial, please visit https://clinical-trials.otsuka.com/. For all approved IPD access requests, Otsuka will share anonymized IPD on a remotely accessible data sharing platform.
