# Supplementary material for: Estimating risk of rapid disease progression in pediatric patients with autosomal dominant polycystic kidney disease: a randomized trial of tolvaptan
Source: Pediatr Nephrol. 2023 Dec 13;39(5):1481–90. doi: 10.1007/s00467-023-06239-8 (PMC10942936; doi:10.1007/s00467-023-06239-8)
Supplement: Supplementary file 1 — Graphical abstract (PPTX 83.1 KB) [file 467_2023_6239_MOESM1_ESM.pptx]

## Slide 1
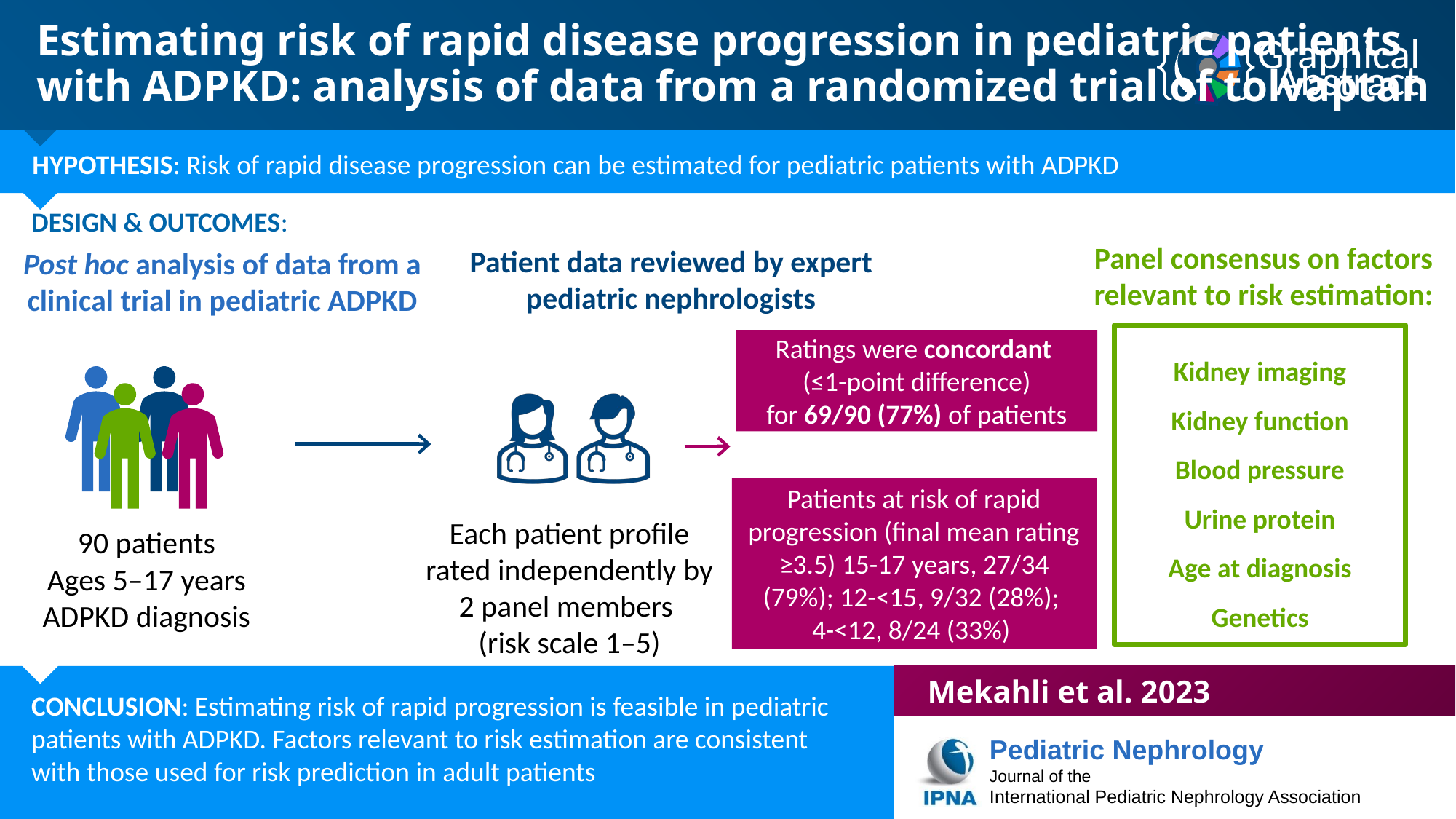

Estimating risk of rapid disease progression in pediatric patients
with ADPKD: analysis of data from a randomized trial of tolvaptan
HYPOTHESIS: Risk of rapid disease progression can be estimated for pediatric patients with ADPKD
DESIGN & OUTCOMES:
Panel consensus on factors relevant to risk estimation:
Patient data reviewed by expert pediatric nephrologists
Post hoc analysis of data from a
clinical trial in pediatric ADPKD
Kidney imaging
Kidney function
Blood pressure
Urine protein
Age at diagnosis
Genetics
Ratings were concordant
(≤1-point difference)
for 69/90 (77%) of patients
Patients at risk of rapid progression (final mean rating ≥3.5) 15-17 years, 27/34 (79%); 12-<15, 9/32 (28%);
4-<12, 8/24 (33%)
Each patient profile rated independently by 2 panel members
(risk scale 1–5)
90 patients
Ages 5–17 years
ADPKD diagnosis
Mekahli et al. 2023
CONCLUSION: Estimating risk of rapid progression is feasible in pediatric patients with ADPKD. Factors relevant to risk estimation are consistent with those used for risk prediction in adult patients
