# Supplementary material for: Estimating risk of rapid disease progression in pediatric patients with autosomal dominant polycystic kidney disease: a randomized trial of tolvaptan
Source: Pediatr Nephrol. 2023 Dec 13;39(5):1481–90. doi: 10.1007/s00467-023-06239-8 (PMC10942936; doi:10.1007/s00467-023-06239-8)
Supplement: Supplementary file 2 — Supplementary file2 (DOCX 33.5 KB) [file 467_2023_6239_MOESM2_ESM.docx]

**Estimating risk of rapid disease progression in pediatric patients with ADPKD: a randomized trial of tolvaptan**

**Online Resource 1**

*Pediatric Nephrology*

Djalila Mekahli, MD, PhD; Lisa M. Guay-Woodford, MD; Melissa A. Cadnapaphornchai, MD; Stuart L. Goldstein, MD; Ann Dandurand, MD;
Huan Jiang, PhD; Pravin Jadhav, PhD; Laurie Debuque, RN

Corresponding author:

Djalila Mekahli, MD, PhD

Department of Pediatric Nephrology

University Hospitals Leuven

Herestraat 49

B-3000 Leuven, Belgium

Email: djalila.mekahli@uzleuven.be

**Online Resource 1.** Examples of cases requiring panelist discussion on how to handle data issues

| **Data Issue** | **Initial Ratings** | **Secondary Rating** | **Resolution of Issue** | **Initial Ratings Adjusted?** | **Final Weighted Rating** |
| --- | --- | --- | --- | --- | --- |
| Disparate volumes between kidneys at screening and baseline in a 5-year-old male  Left kidney: 270/231 mL at screening/baseline  Right kidney: 110/118 mL at screening/baseline  Month 12 data showed a similar disparity | 1 and 4 | 5 | All reviewers agreed that kidney volume disparity was consistent over time. Presence of borderline hypertension, large total kidney volume, and hyperfiltration all supported rating of high risk  Reviewers agreed that the disparity should therefore not impact the rating | The 1 was changed to a 4 during the panel discussion | 4.4 |
| Wide range of eGFR values (in mL/min/1.73 m^2^) at screening (97), baseline (59), and during the study (44 to 101). Male aged ≥4 to <12 years | 2 and “either a 2 or a 5” | n/a  (due to indeterminate initial rating) | All reviewers agreed that such eGFR fluctuations do occur and are dependent on hydration and tolvaptan dosing  Reviewers agreed that the disparity should therefore not impact the rating | The indeterminate initial rating was changed to a 3 during the panel discussion | 2.5 |
| Missing kidney volume imaging data at baseline in a 9-year-old female | 1 and “?” | n/a  (due to missing baseline kidney volume data) | Month 12 kidney length data were available and used to estimate total kidney volume (~400 mL)  Given the estimated volume and presence of hyperfiltration and borderline hypertension, the panelists agreed the subject was at high risk for progression | The indeterminate initial rating was changed to a 1 during a pre-panel discussion | 1 |
| Renal pelvis measurements appeared to be incorrect in a 15-year-old male (left: 12.3 cm; right: 13.6 cm) at screening; may have actually been kidney length | 3 and 4 | n/a (concordant initial ratings) | Total kidney volume on MRI at screening was available (496 mL) and the panelists agreed that renal pelvis measurements would not affect risk estimation | No | 3.5 |
| Kidney width but not length was available for a female aged 6 years and in CKD stage G2 | 4 and 1 | 5 | All panelists agreed that lack of a kidney length measurement would not impact the rating, given the reduction in kidney function | No | 3.6 |

Risk progression rating range from 1 (least risk) to 5 (highest risk).

CKD, chronic kidney disease; eGFR, estimated glomerular filtration rate.
